# The Role of Hormones in Hidradenitis Suppurativa: A Systematic Review

**DOI:** 10.3390/ijms232315250

**Published:** 2022-12-03

**Authors:** Nessr Abu Rached, Thilo Gambichler, Johannes W. Dietrich, Lennart Ocker, Caroline Seifert, Eggert Stockfleth, Falk G. Bechara

**Affiliations:** 1 International Centre for Hidradenitis Suppurativa/Acne Inversa (ICH), Department of Dermatology, Venereology and Allergology, Ruhr-University Bochum, 44791 Bochum, Germany; 2 Diabetes, Endocrinology and Metabolism Section, Department of Internal Medicine I, St. Josef Hospital, Ruhr University Bochum, NRW, Gudrunstr. 56, 44791 Bochum, Germany; 3 Diabetes Centre Bochum-Hattingen, St. Elisabeth-Hospital Blankenstein, Im Vogelsang 5-11, 45527 Hattingen, Germany; 4 Centre for Rare Endocrine Diseases, Ruhr Centre for Rare Diseases (CeSER), Ruhr University Bochum and Witten/Herdecke University, Alexandrinenstr. 5, 44791 Bochum, Germany; 5 Centre for Diabetes Technology, Catholic Hospitals Bochum, Gudrunstr. 56, 44791 Bochum, Germany

**Keywords:** hidradenitis suppurativa, acne inversa, hormones, spironolactone, metformin, finasteride, insulin resistance, thyroid function, endocrinology, adipokine

## Abstract

Hidradenitis suppurativa (HS) is a chronic inflammatory disease manifesting in inverse body regions. In a systematic review, the role of hormones in HS will be presented to better understand the pathomechanisms of HS. The review is based on the PRISMA criteria. Systematic research was carried out using keywords. Subsequently, the data were analyzed based on the clinical response and other relevant information. The main focus of our systematic review was on HS manifestation, exacerbation, sex hormones, antiandrogen therapy, thyroid function, polycystic ovary syndrome, insulin resistance, and adipokines. In HS, there appears to be a dysregulated adipokine release that is shifted towards pro-inflammatory adipokines. Insulin resistance is significantly more common in HS than in healthy patients regardless of BMI, age, and gender. Insulin resistance in HS patients leads to further cardiovascular disease. The mechanism of insulin resistance and role of adipokines should be investigated in future studies to better provide the pathomechanisms of HS. The role of androgens seems to be important in a certain subgroup of female patients. Anti-androgenic therapy can be useful and helpful in some patients. However, further studies are needed to better understand the hormonal relationship in HS.

## 1. Introduction

Hidradenitis suppurativa (HS), also known as acne inversa, is a chronic, inflammatory skin disease of inverse body regions. HS is a globally underestimated disease with a prevalence of 0.4–1% [1,2]. Some data describe a prevalence of 4% [3]. The disease is accompanied by inflammatory nodules, abscesses, scars, contractures, and fistulas that significantly reduce the patient’s quality of life. Therapy of HS consists of local antiseptics, local antibiotics, systemic antibiotics, high-dose zinc therapy, biologics, and surgical interventions [4]. In advanced disease with many fistulas, surgery and combined immunomodulatory therapies can be indicated. In some cases, preoperative downstaging with antibiotics may be useful. There is currently no curative therapy as the pathogenesis is not yet fully understood. According to current knowledge, the pathogenesis of HS is multifactorial. HS is associated with smoking, obesity, and metabolic syndrome. Use of hormonal drugs for treatment of HS is discussed in the literature. In some cases, taking medications such as spironolactone, which also have an anti-hormonal effect, leads to an improvement in HS [5,6].

The hormonal role in the pathogenesis of HS is not yet fully understood. A worsening of HS is often described by patients premenstrually and after pregnancy [7]. Therefore, a hormonal change could be a cause of HS. Many patients with HS have impaired glucose tolerance and insulin resistance. There are some case control studies describing an improvement in HS under antidiabetic or antiandrogenic therapy. The aim of this work is to summarize the current knowledge regarding hormones and their possible role in the molecular pathomechanisms of HS.

## 2. Methods

**Figure 1:**
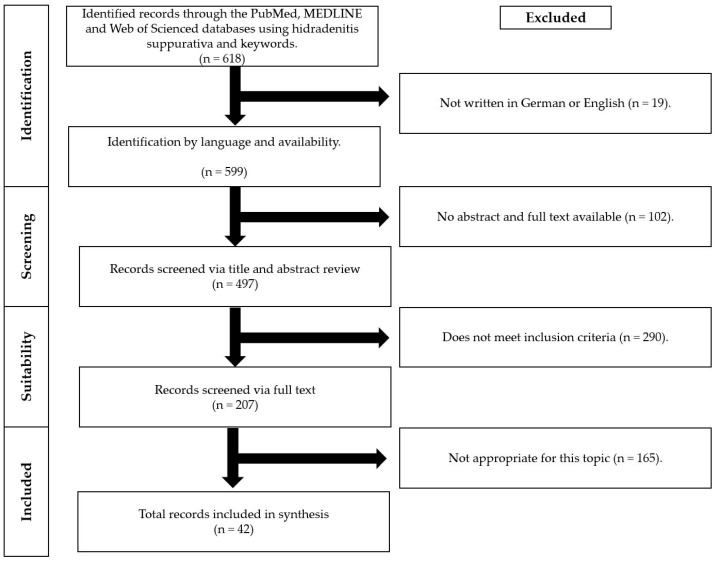
Flowchart with the research strategy in this systematic review.

In this work, a systematic review has been conducted for articles on PubMed, MEDLINE, and Web of Science databases published between 1985 and 2022. This work has been conducted according to the PRISMA criteria [8]. The main focus of this research was on recent data of the last five years. Only articles in English and German have been included in this review. The flow chart with the research strategy can be seen in Figure 1. Meta-analyses, randomized double-blind trials, experimental studies, case-control studies, cross-sectional studies, and retrospective chart reviews were included for the review. Recent case reports about hormones have also been mentioned in this review so that new trends can be considered. Reviews, abstracts, and letters to the editor were not included. The screening for suitability and selection of the records has been carried out by two persons. In case of disagreement about inclusion, a third person was consulted. The following search terms were used: (“hidradenitis suppurativa” OR “acne inversa”) AND (“hormones” OR “hormone” OR “pregnancy” OR “menstruation” OR “menopause” OR “puberty” OR “finasteride” OR “spironolactone” OR “flutamide” OR “CPA” OR “cyproterone acetate “ OR “metformin” OR “insulin resistance” OR “insulin” OR “IGF-1” OR “insulin-like-growth-factor-I” OR “somatomedin-C” OR “HOMA-IR” OR “glucose metabolism” OR “pancreas” OR “beta cells” OR “adipocytes” OR “adipokine” OR “visfatin” OR “resistin” OR “leptin” OR “RBP4” OR “Retinol binding protein 4” OR “adiponectin” OR “omentin-1” OR “metabolic syndrome” OR “obesity” OR “diabetes” OR “weight loss” OR “bariatric surgery” OR “antidiabetic” OR “endocrinology” OR “endocrine” OR “PCOS” OR “polycystic ovary syndrome” OR “thyroid function” OR “thyroid” OR “antiandrogens” OR “sexual hormones” OR “androgens” OR “androgen” OR “testosterone” OR “estrogen” OR “progesterone” OR “LH” OR “FSH” OR “trigger factors” OR “pituitary gland” OR “hypothalamus” OR “pituitary-hypothalamus axis” OR “hormonal balance”). Included articles were screened for suitability based on the title and abstract. Subsequently, the original data and information of the full texts were evaluated. The references of identified records have been used to expand the search. In addition, we searched for current clinical trials in the library clinical trials using the keyword HS.

## 3. Results

In this systematic review, 42 articles have been included. The included articles dealt with “HS manifestation and exacerbation” (7%), “sexual hormones” (14%), “anti-androgen therapy” (29%), “thyroid function” (21%), “polycystic ovary syndrome” (5%), and “insulin resistance and adipokines” (24%). Clinical response has been assessed using Hidradenitis Suppurativa Physician’s Global Assessment (HS-PGA), Hurley system, Dermatology Life Quality Index (DLQI) [9], or/and International Hidradenitis Suppurativa Severity Score System (IHS4) [10] in the studies.

### 3.1. HS Manifestation and Exacerbation

The hormonal influence on HS is evident in the typical fluctuations of the disease. HS usually begins peripubertally and shows exacerbation after pregnancy (Table 1). Detailed analysis of the timing from the fluctuations could provide further insight into HS pathogenesis. Perimenstrually, 43–76.7% of patients report subjective worsening in HS [7,11]. A meta-analysis has shown that about 25% of the patients report improvement and 20% of the patients report worsening in HS during pregnancy [12]. However, about 60% of the patients show worsening in HS postpartum [12]. All these results support the hormonal influence in HS.

### 3.2. Sexual Hormones

It has long been suspected that pathophysiology of HS is linked to sex hormones. Sex hormones are steroid hormones that are produced in the gonads, placenta, or adrenal cortex. Hormones are especially important for human reproduction and formation of the sex. The release is controlled by the hypothalamic-–pituitary axis. The male sex hormones include androgens and female sex hormones include estrogens, gestagens, and hCG. The connection between HS and sex hormones is obvious, but, so far, the exact connection could be not clarified. HS shows clinical deterioration or improvement at certain times, characterized by hormonal fluctuations. During pregnancy, clinical improvement may occur in HS. The frequent peripubertal onset of HS and perimenstrual flares suggest that sex hormones are involved in HS pathogenesis (Table 2).

In an experimental study, immunohistochemical expression of the androgen receptor (AR) and estrogen receptor (ER) in HS skin tunnels has been investigated [13]. HS showed increased AR expression in the infundibulum and skin tunnel compared to healthy skin. AR expression was higher in males than in females. Immunohistochemical ER expression was predominantly negative. In a microarray analysis of gene expression, HS lesions showed increased androgen receptor (AR) transcriptional activity compared to non-lesional skin [14]. Apocrine glands in HS show no difference in AR and ER receptor expression [15]. Zouboulis’ study also showed a subordinate role of the apocrine gland in HS [16]. Interestingly, androgen-controlled genes were up-regulated in women, while genes affecting fat metabolism were down-regulated in men [16]. Mortimer et al. discovered early on that female HS patients have an elevated testosterone and free androgen index [17]. There are three cases reported where cross-sex hormone therapy (CSHT) with testosterone resulted in an exacerbation or manifestation of HS [18,19]. In contrast to the above results, Harrison reports that there is no difference in testosterone between HS and healthy controls [20]. After stimulation by thyrotropin-releasing hormone and gonadotropin-releasing hormone, increased values were found for prolactin and TSH [20]. This is remarkable as usually the response to TRH testing is blunted in males compared to women [21,22]. Harrison et al. concluded that there might rather be a disturbance in the feedback signals of the peripheral hormones in HS. There are no recent data on the hypothalamic–pituitary axis. The available data on antiandrogenic therapy in HS are analyzed in the next chapter.

**Table 2:** Current findings about sexual hormones in HS.

References	Type of Source	Aim	Results	Conclusion
Harrison et al., 1985 [20]	case-control study (n = 13; all female)	Investigation of endocrine abnormality in HS	− by adding thyrotropin-releasing hormone and gonadotropin-releasing hormone, the HS prolactin and TSH responses were significantly higher than in controls − there was no difference in the levels of estrogen, progesterone, testosterone, DHEA-S, T3, and T4.	In HS, there could rather be a disturbance of the feedback signals of peripheral hormones.
Mortimer et al., 1986 [17]	case-control study (n = 42; all female)	role of sexual hormones in HS	− 13 patients (31%) had irregular menstruation, 22 out of 36 (61.1%) had exacerbation of HS premenstrual − patients had higher testosterone (*p* < 0.01) and free androgen index (testosterone to sex-hormone-binding globulin concentrations) than healthy controls (*p* < 0.01) − 88.1% of the patients had comedones in typical HS area.	In female patients with follicular type of HS, androgens seem to play a role in the pathogenesis.
Buimer et al., 2015 [15]	experimental im-munohisto-chemical study (n = 22; 16 female and 6 male)	AR and ER immunoreactivity in apocrine glands of HS	− expression of ER in the apocrine glands was weak or absent − AR expression in the apocrine glands was strong, but there was no difference between HS and healthy controls	Expression of AR and ER in apocrine glands has no decisive role in the pathogenesis of HS.
Gauntner 2019 [14]	experimental study (n = 17; gender unknown)	gene expression microarray analysis from skin biopsies in HS	− genes (VDR, NFE2L2, IRF8, RELA, NANOG, AR, TP63, STAT3) were significantly more enriched in lesional skin of HS compared to non-lesional skin.	HS lesions show increased androgen receptor (AR) transcriptional activity, activation of stem-cell-associated transcriptional pathways, and upregulation of Notch-associated genetic loci.
Zouboulis et al., 2020 [16]	experimental study (n = 16; 8 female and 8 male)	Transcriptome analysis to investigate the role of the apocrine glands in HS	− in female patients, the genes MRO, DYRK3, SDK2, GLB1L, CATSPERB, and PRPS2 are upregulated. All the genes mentioned are regulated by androgens. − In male patients, the genes (AGPAT3, GAL, ELOVL3, THRSP, DGAT2L3, OLAH, THRSP, FADS1, NR2F2, FADS2, PTGDS, and HAO2) that play a role in fat metabolism are downregulated − the gene SULF1 showed significant upregulation between lesional skin (LS) and non-lesional skin (NLS)	Androgen-controlled genes are up-regulated in women, and genes that influence fat metabolism are down-regulated in men. The apocrine glands are of secondary importance in HS pathogenesis.
Yu et al., 2021 [13]	experimental immunohistochemical study (n = 10; 6 female and 4 male)	AR immunoreactivity in HS skin tunnels, epidermis, and infundibulum	− AR expression in the epidermis and infundibulum was on average higher in HS than in healthy skin (54% vs. 26%) − AR expression was significantly higher in HS skin tunnels (lining epithelium) in both males and females − Males showed higher AR expression than females (average: 226% and 78%) − Males had an extensive, continuous AR expression pattern, while females had a segmental, patchy pattern − estrogen receptors were predominantly immunohistochemically negative in the epidermis and skin tunnels	Androgens are involved in HS. The segmental AR expression pattern of the skin tunnels in women may be related to a response to antiandrogenic therapy.

### 3.3. Anti-Androgen Therapy

The efficacy of different drugs with an anti-androgenic effect further supports the role of hormones in HS (Table 3, Table 4 and Table 5). Clinical improvement has been observed in female HS patients with the use of antiandrogenic drugs. In this paper, anti-androgenic drugs finasteride, spironolactone, and cyproterone acetate have been analyzed in HS.

In a retrospective study by Kraft et al., 64 female patients with HS were included. Antiandrogenic therapy was more effective compared to antibiotic therapy (55% vs. 26%; *p* < 0.04) [23]. These positive results were included in the 2019 North American guideline for HS. They recommend use of hormonal agents in appropriate patients and mild to moderate HS [24]. Estrogen-containing oral contraceptives, spironolactone, cyproterone acetate, metformin, finasteride, and flutamide should be used in combination with other agents [24].

There are some case series that show a positive effect of finasteride in HS [25,26,27,28]. Finasteride is a competitive inhibitor of steroid 5α-reductase and has an anti-androgenic effect. Conversion of testosterone into dihydrotestosterone (DHT) is selectively inhibited by finasteride. DHT is the biologically active form of testosterone. Testosterone circulates in blood and enters the cell through lipophilic character [29]. Within the cell, it is converted into DHT. DHT and the intracellular androgen receptor form a hormone–receptor complex that migrates into the cell nucleus and binds to a hormone-responsive element (HRE) [29]. This interaction thus influences gene expression. In Germany and the USA, finasteride is approved in a daily dose of 1 mg for androgenic alopecia and 5 mg for benign prostatic hyperplasia [30]. In all four reports, the patients showed a significant improvement in HS [25,26,27,28]. Under therapy with finasteride, there was a decrease in frequency and intensity of HS relapses. In some patients, use of finasteride resulted in complete healing of lesions. In most cases, a daily dose of 5 mg was used and well-tolerated by the patients. In rare cases, headache, nausea, menstrual irregularities, breast tenderness, or decreased libido/sexual function have been described as adverse events [27]. It should be noted that all case series reported predominantly about female patients, so adverse events could be significantly more severe in male patients [31]. Overall, finasteride appears to be a safe and effective treatment option for HS.

**Table 3:** The use of finasteride (antiandrogen therapy, inhibitor of steroid 5α-reductase) in HS.

References	TYPE OF SOURCE	Dose	Results	Conclusion
Joseph et al., 2005 [25]	case series in adults (n = 7; 5 female and 2 male)	5 mg/d as monotherapy	− six patients (85.7%) showed significant improvement, and, in three of them (42.9%), the lesions healed completely − two women (28.6%) showed breast enlargement under the therapy; otherwise, therapy was well-tolerated by all of them	Finasteride is an effective therapeutic option in HS
Randhawa et al., 2013 [28]	case series in children with HS (n = 3; all female)	5 mg/d as monotherapy	− oral finasteride decreased frequency and severity of disease flares with no significant adverse effects	Finasteride is a therapeutic option for children
Mota et al., 2017 [26]	case series in children with HS (n = 5; 4 female and 1 male)	1 to 5 mg/d as monotherapy	− a general improvement in the disease was observed in all patients, with a decrease in the frequency and intensity of the relapses − no adverse effects have been observed	Use of finasteride in AI may be a treatment option in children
Babbush et al., 2022 [27]	retrospective chart review of female patients (n = 20; all female)	5 mg/d as monotherapy	− 10 out of 20 patients (50%) reported that finasteride showed a treatment response, 7 patients (35%) were neutral, and 3 patients (15%) were dissatisfied with the treatment response − 80% of patients (n = 16) showed no adverse effects from finasteride − 20% of patients (n = 4) reported side effects: headache, nausea, menstrual irregularities, breast tenderness, or decreased libido/sexual function	Finasteride is a safe and effective alternative for female patients with HS

In recent years, use of spironolactone in young female patients has increased significantly [32]. In the age group of 13 to 19 years, its usage has increased three times compared to previous years [32,33]. Spironolactone is a competitive aldosterone receptor antagonist and has weak antiandrogenic, estrogenic, and glucocorticoid effects. Spironolactone is a potassium-sparing diuretic and is licensed for treatment of severe heart failure, resistant hypertension, nephrotic syndrome, liver cirrhosis, and Conn’s syndrome [34]. The response to spironolactone in HS ranges from 42 to 85% [5,35]. There was no difference between monotherapy with spironolactone and combination with other drugs [36]. Spironolactone can be administered in a dose of 25 to 200 mg [5,6,35,37]. A daily dose of 100 mg has been administered most frequently. All reports of antiandrogenic therapy with spironolactone in HS are from female patients. Spironolactone also has a positive effect on quality of life [37]. A randomized phase 4 trial was designed to investigate the efficacy and optimal dose of spironolactone in HS (ClinicalTrials.gov Identifier: NCT04100083). The study was discontinued due to lack of funding.

**Table 4:** Use of spironolactone (antiandrogen therapy, aldosterone antagonist) in HS.

References	Type of Source	Dose	Results	Conclusion
Lee et al., 2015 [5]	case series of 20 women (n = 20; all female)	100 to 150 mg/d as monotherapy ± minocycline ± cyproterone acetate	− 17 out of 20 patients (85%) showed a response to therapy after 3 months with a reduction in HS-PGA − 11 out of 20 patients (55%) achieved complete disappearance of the skin lesions − there was no difference between monotherapy and combination therapy	Spironolactone should be considered as first-line treatment in women
Golbari et al., 2019 [6]	single-center retrospective study (n = 67; all female)	25 to 200 mg/d as monotherapy ± antibiotics ± contraceptives	− spironolactone significantly reduces lesion count, HS-PGA score, and pain − no significant change stage or fistulas − there was no difference in improvement between a dose less than 75 mg and more than 100 mg	Spironolactone reduces lesion count, HS-PGA score, and pain
McPhie et al., 2019 [35]	retrospective chart review (n = 12; gender unknown)	100 mg/d ± adalimumab ± isotretinoin or/and doxycycline	− 42% (5 out of 12) of the patients had an improvement in IHS4 score − 50% had no change and 8% had a worsening of the score.	Combination therapy, e.g., with spironolactone, can be useful
Quinlan et al., 2020 [37]	retrospective study (n = 26; all female)	monotherapy with 50 to 100 mg/d	− spironolactone reduced the DLQI> 5 (35%) in nine patients − no comment on further clinical data	Spironolactone appears to be a treatment option for women with HS
Horissian et al., 2022 [32]	epidemiological cross-sectional analysis (n = 215; all female)	dose unknown	− between 2014 and 2018, use of spironolactone in HS increased two- to threefold	Increased use of spironolactone is likely driven by the successful treatment.

One of the first hormonal drugs used in HS is cyproterone acetate (CPA). CPA is a competitive antagonist at the androgen receptor and agonist at the progesterone receptor. CPA prevents testosterone and dihydrotestosterone from binding to the receptor. CPA also lowers serum levels of gonadotropins LH and FSH. Further, 21-hydroxylase is also inhibited by CPA so that synthesis of mineral corticoids (e.g., aldosterone) or glucocorticoids (e.g., cortisol) is inhibited. There are positive reports regarding use of CPA in HS [23,38,39]. In a randomized trial, CPA has been compared with norgestrel [38]. There was no significant difference between the two groups. Both drugs showed a similar response.

**Table 5:** Use of cyproterone acetate (CPA, competitive antagonist at the androgen receptor) in HS.

References	Type of Source	Dose	Results	Conclusion
Sawers et al., 1986 [39]	case series (n = 4; all female)	CPA 100 mg/d + 30 to 50 µg/d ethinyl oestradio	− 75% of patients had vulvar HS involvement − 100% of patients showed rapid objective clinical and subjective improvement − after reduction of CPA to 50 mg/d, 75% of patients showed worsening	In HS, there is an androgen-dependent disorder. Combined cyproterone acetate and estrogen therapy in women may be useful.
Mortimer et al., 1986 [38]	randomized double-blind crossover trial (n = 24; all female)	CPA 50 mg/d + 50 µg/d ethinyl oestradio	− 71% of patients had vulvar HS involvement − 50% showed improvement in their disease and 30% showed complete remission over 18 months	Anti-androgen therapy may be beneficial in HS.
Kraft et al., 2007 [23]	retrospective chart review (n = 29; all female)	CPA 2 to 25 mg/d + 35 µg/d ethinyl oestradio ± spironolactone 100 mg/d	− 55% of the patients showed an improvement in the HS symptoms.	Hormone treatment should be considered for women.

Flutamide has a non-steroidal anti-androgenic effect. One case report reports an improvement in HS under flutamide with a dose of 250 mg daily [40]. The antidiabetic drug metformin has a mild anti-androgenic effect and is used in PCOS. The data on metformin and HS are listed in the subsection on insulin resistance and adipokines.

### 3.4. Insulin Resistance and Adipokines

An association between HS and metabolic syndrome has been reported in several studies [41]. Truncal obesity, hypertension, diabetes mellitus type II, and dyslipoproteinemia, characteristics of metabolic syndrome, are frequently found in HS patients. Metabolic syndrome is present in 32.4% of HS patients [42]. A recent meta-analysis estimated the pool ratio of metabolic syndrome in HS to be 2.66 (95% CI: 1.90–3.72) [43]. There is also an association between HS and diabetes mellitus [44,45]. A meta-analysis showed that there is a 1.69-fold increased risk of developing diabetes mellitus in HS [46]. A cross-sectional analysis in the United States showed an overall prevalence of 24.8% for type 2 diabetes mellitus in HS [47]. In the same cohort, the prevalence of type 2 diabetes mellitus in non-HS patients was 15.6%. In a comparative cross-sectional study of 3207 patients, a significant association was presented between HS and metabolic syndrome [odds ratio (OR) 1.61, 95% confidence interval (CI) 1.36–1.89], diabetes mellitus (OR 1.41, 95% CI 1.19–1.66), obesity (OR 1.71, 95% CI 1.53–1.91), hyperlipidemia (OR 1.14, 95% CI 1.02–1.28), and hypertension (OR 1.19, 95% CI 1.03–1.38) [48]. Many patients have impaired glucose tolerance. The exact mechanism of impaired tolerance is not clear. 

There is some research on adipokine levels in HS *(*Table 6). Adipokines are signaling molecules produced by adipose tissue. They act as a link between the immune system and energy metabolism. With weight gain, the pro-inflammatory effect increases, and, with hunger, the anti-inflammatory effect increases. Known adipokines include plasminogen activator inhibitor-1 (PAI-1), leptin, visfatin, adiponectin, apelin, interleukin-6 (IL-6), monocyte chemotactic protein-1 (MCP-1), retinol binding protein 4 (RBP4), TNF-α, omentin-1, and vaspin. Secretion and expression of adipokines is disturbed in cardiovascular diseases and obesity [49]. In a case-control study by Malara et al., the adipokines adiponectin, resistin, and leptin in blood were compared to healthy controls, nondiabetic obese group, and psoriasis patients [50]. The mean serum levels of adiponectin were significantly decreased in patients with HS compared to healthy lean controls, and the resistin and leptin levels were increased [50]. In all the patient groups, BMI and serum levels of leptin (r = 0.83) and resistin (r = 0.6) were strongly correlated. The authors suggested that serum levels of adipokines are dysregulated in HS and are associated with obesity [50]. Expression of adipokines in this study was shifted towards pro-inflammatory resistin and leptin. Akdogan et al. found that serum levels of visfatin after adjustment for BMI and smoking status differed significantly (*p* = 0.02) and increased the risk of HS 1.56-fold [51]. González-López also reports that visfatin and resistin are independent risk factors for HS [52]. Both adipokines were independent of age, sex, and body mass index. Adiponectin was inversely associated with IR (OR 0.994; CI 95%, 0.989–0.999; *p* = 0.023) and resistin positively (OR 1.012; CI 95%, 1.001–1.024; *p* = 0.03) after adjustment (age, sex, BMI, and smoking status) [52]. However, there seems to be no correlation between serum levels of adipokines and severity of HS [52]. Another adipokine is retinol binding protein 4 (RBP4), which is produced by fat cells [53]. RBP4 is elevated in insulin resistance and also in diabetes mellitus [54,55]. RBP4 is also a transport protein for free vitamin A [56]. In a cross-sectional study of 137 patients (77 HS patients and 60 controls) without diabetes mellitus, higher RBP4 and lower ghrelin levels were found in HS [57]. RBP4 levels correlated positively with disease severity and insulin resistance in HS patients independent of BMI [57]. There was no correlation between disease severity and ghrelin. Omentin-1 was also found to be elevated in HS patients compared to healthy controls after adjustment for BMI, age, and sex [58]. Omentin-1 is an adipocytokine expressed in visceral fat. Omentin-1 has an important role in body metabolism and insulin sensitivity via AMP-activated protein kinase [59].

**Figure 2:**
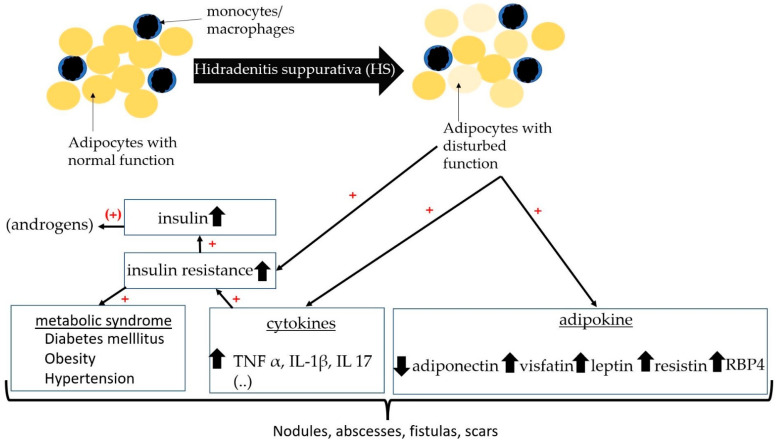
Schematic diagram with relationship between insulin resistance, adipokines, and HS.

Increased incidence of insulin resistance in HS also suggests a disturbed hormonal axis [42,51,60]. Insulin resistance has often been determined by the Homeostatic Model Assessment for Insulin Resistance (HOMA-IR). HOMA-IR is calculated from the product of fasting insulin (µu/mL) and fasting blood glucose (mg/dL), which is then divided by 405 [61]. A HOMA-IR value above 2.5 may indicate insulin resistance. In a cross-sectional and case-control study, 76 patients with HS and 61 age- and sex-matched control subjects have been compared for the presence of insulin resistance [60]. The median HOMA-IR value was significantly higher in HS than in controls (2.0 vs. 1.5; *p* = 0.01) [60]. Prevalence of IR was also significantly higher than in controls (43.4% vs. 16.4%; *p* = 0.001) [60]. The authors, therefore, recommend that HS patients should be screened for IR. The study by Akdogan et al. and Özkur et al. also showed a significant correlation between HS and insulin serum levels [42,51]. Weight loss in HS patients has a positive effect on insulin resistance and can lead to a decrease in HS lesions and insulin resistance [62]. This supports the central role of insulin resistance in HS (Figure 2).

**Table 6:** Current findings about adipokines and insulin resistance in HS.

References	Type of Source	Aim	Results	Conclusion
Malara et al., 2018 [50]	case-control study (n = 30; gender unknown) compared to psoriasis, nondiabetic obese (BMI > 30), and lean control groups (BMI < 25)	analysis of adipokines (adiponectin, resistin, and leptin) in serum in HS	− serum level of adiponectin was decreased significantly in HS in comparison with healthy lean controls − adiponectin was decreased significantly in the nondiabetic obese group compared with the psoriasis group and healthy lean controls − resistin and leptin were increased significantly in HS compared with healthy controls	Serum levels of adipokines are dysregulated in HS and are associated with obesity.
Vilanova et al., 2018 [60]	cross-sectional, case-control study (n = 76; 39 female and 37 male)	aim was to analyze the prevalence of insulin resistance (IR) in patients with HS	− median homeostasis model assessment of IR (HOMA-IR) value in HS patients was significantly higher than in controls (*p* = 0.01; irrespective of age, sex, and BMI)	Increased frequency of IR in HS.
Akdogan et al., 2018 [51]	case-control study (n = 40; 17 female and 23 male)	aim was to evaluate serum visfatin levels (SVLs), insulin levels (SILs), and insulin resistance (IR) in HS	− SVLs, SILs, and IR were significantly higher in HS (*p* = 0.02, *p* = 0.001, *p* < 0.001)	HS patients have higher SVL, SIL, and IR values than healthy controls—independent of BMI and smoking status.
González-López et al., 2020 [52]	case-control study (n = 76; 40 female and 36 male)	aim was to analyze serum concentrations of adiponectin, leptin, resistin, and visfatin in non-diabetic patients with HS	− serum adiponectin concentrations were significantly lower, and resistin and visfatin levels were significantly higher in HS patients than in controls (irrespective of age, sex, and BMI) − no association between serum levels of adipokines and HS severity	Adipokines might play a role in development of insulin resistance.
González-López et al., 2020 [57]	cross-sectional, case-control study (n = 77; 40 female and 37 male)	aim was to determine the serum levels of Retinol binding protein 4 (RBP4) and ghrelin in HS, and to assess the relationship between these levels and IR, disease severity, and HS risk	− RBP4 is higher, and ghrelin is lower in HS than controls − RBP4 levels were positively correlated to disease severity and IR in HS − no association between ghrelin levels and any clinical parameters − high serum RBP4 and low ghrelin were associated with an increased risk for HS.	High RBP4 levels may be a surrogate biomarker for IR in patients with HS.Increased RBP4 and decreased ghrelin levels could also be independent risk factors for development of HS.
Özkur et al., 2020 [42]	case-control study (n = 37; 28 female and 9 male)	aim was to evaluate serum irisin, plasma glucose, insulin, and lipid levels in HS	− Insulin resistance was higher in HS patients than in controls (45.9% vs. 8.1%; *p* = 0.003) − plasma triglycerides, glucose, and insulin levels were significantly higher in HS (*p* = 0.013, *p* = 0.001, and *p* = 0.004) − irisin level was insignificantly higher in HS	Patients with HS should be screened for insulin resistance and metabolic syndrome.
González-López et al., 2021 [58]	case-control study (n = 78; 41 female and 37 male)	aim was to investigate serum omentin-1 and apelin levels in non-diabetic patients with HS	− serum omentin-1 was significantly higher in HS (irrespective of age, sex, and BMI) − apelin serum levels were not significantly different − no association between serum levels of both omentin-1 and apelin with HS severity	Patients with HS have raised omentin-1 serum levels.

Metformin is an oral antidiabetic drug that increases the effect of insulin. The insulin sensitizer has the effect of reducing glucose production in liver and increasing glucose utilization by muscle and fat cells. Metformin also has an anti-inflammatory effect on several cell types. It has been reported by Chung et al. that metformin reduces production of nitric oxide (NO), prostaglandin E2 (PGE2), and pro-inflammatory cytokines, such as IL-6, IL-1β and TNF-α [63]. This is probably reduced by inhibiting activation of the nuclear factor kappa-light-chain-enhancer of activated B cells (NF-κB) in macrophages. A therapeutic response of metformin in HS patients has already been described in the literature *(*Table 7*)*. In a prospective study (n = 25) with metformin, 72% of the patients experienced clinical improvement in HS [64]. DLQI also improved in 64% of the patients. In a retrospective study of children with HS, response to therapy was 50% of the patients [65]. In Jennings’ study, most patients on metformin therapy have been analyzed so far [66]. The study showed a treatment response of 68 %. In 19% of the patients, use of metformin led to complete remission. It was interesting to note that 75% of the patients had insulin resistance [66]. Overall, metformin has been well-tolerated by patients. The most common side effect was gastrointestinal symptoms [64,65,66]. The effect of metformin on HS is currently being investigated in the first double-blind randomized trial called “Rediscovery of Metformin in Chronic Invalidating Autoinflammatory Disease Hidradenitis Suppurativa” (ClinicalTrials.gov Identifier: NCT04649502). The primary endpoint of the metformin study is clinical response, assessed by IHS4. Secondary endpoints include insulin resistance, lesion count, pain, HS-PGA, cost-effectiveness, change in biomarker calprotectin, relapses, treatment satisfaction, DLQI, safety, and tolerability. In the first study arm, patients receive metformin and doxycycline. In the placebo arm, patients receive placebo and doxycycline. The first results of the metformin trial are expected in 2023.

Another antidiabetic drug is liraglutide. There is only one case report of clinical experience with liraglutide in HS [67,68]. Liraglutide is a GLP-1 agonist and directly stimulates GLP-1 receptor. Stimulation of GLP-1 results in glucose-dependent insulin secretion and inhibition of glucagon secretion. As with metformin, most common side effects of liraglutide include gastrointestinal symptoms. In rare cases, pancreatitis and pancreatic carcinoma can occur. After four weeks of liraglutide therapy, an obese patient who was not insulin-resistant experienced clinical improvement in HS and DLQI [67]. HS-PGA decreased from 4 to 1 and DLQI from 24 to 14 under liraglutide.

**Table 7:** Current findings about antidiabetic drugs metformin (enhancement of the insulin effect) and liraglutide (Glucagon-like Peptide-1 agonist, GLP-1 agonist) in HS.

References	Type of Source	Drug (Dose)	Results	Conclusion
Verdolini et al., 2013 [64]	prospective study (n = 25; 22 female and 3 male)	Metformin (dose of 500 to 1500 mg)	− 18 patients (72%) showed clinical improvement, with a significant mean reduction in sartorius score (reduction of 12.7) − DLQI showed significant improvement in 16 cases (64%), with a decrease in DLQI score of 7.6 points	Metformin may be a therapeutic option for treatment of HS.
Jennings et al., 2017 [67]	case report (n = 1; female)	Liraglutide (start with 0.6 mg subcutaneously per day, then weekly 1.8 mg)	− After 4 weeks of therapy, HS improved significantly (HS-PGA from 4 to 1). − Liraglutide lowers interleukin-17 and TNF-α-induced cytokines, including nuclear factor-κB. − Liraglutide led to a reduction in DLQI and high weight loss	In obese patients with HS, use of liraglutide may be beneficial.
Moussa et al., 2020 [65]	retrospective chart review with pediatric patients (n = 16; gender unknown)	Metformin (dose unknown)	− 11 patients had Hurley I at the beginning of the study and 5 patients had Hurley II. − 5 patients showed improvement on metformin with a lower frequency of relapses, 5 patients had no improvement, and 6 patients discontinued follow-up or no data were available − 5 out of 10 patients (50%) showed improvement in HS − 2 patients discontinued therapy due to gastrointestinal symptoms	Metformin as add-on therapy can lead to better control of HS in children, with minimal side effects.
Jennings et al., 2020 [66]	retrospective chart review (n = 53; 45 female and 8 male)	Metformin (dose of 500 to 1500 mg)	− subjective clinical response was 68% (n = 36) and 19% of patients (7/36) achieved complete remission on metformin monotherapy. − 25% of patients did not improve − insulin resistance was present in 75% of patients − presence of insulin resistance was not associated with clinical response to metformin.	Metformin is an effective, well-tolerated, and cost-effective treatment for HS.

### 3.5. Thyroid Function in HS

The thyroid gland also plays an important role in human hormone balance. In the thyroid gland, iodine-containing hormones including triiodothyronine (T3) and thyroxine (T4) are produced by thyrocytes. Both hormones are important in metabolic process and growth. T3 is biologically more active than T4 and is produced from T4 by type 1 and 2 deiodinases. Thyroid hormones act on many organs. In general, two hormones have an increasing effect on energy metabolism. Thyroid hormones affect the heart by increasing expression of β-receptors and heart rate [69]. In metabolism, they increase basal metabolic rate and oxygen consumption. Glucose absorption, gluconeogenesis, and glycogen synthesis are increased. Regulation of the thyroid gland takes place via the hypothalamus–pituitary axis. Thyroid-stimulating hormone (TSH) from the pituitary gland is released after stimulation by thyrotropin-releasing hormone (TRH) from the hypothalamus. TSH stimulates T3 and T4 synthesis. In hyperthyroidism, there is increased production of the hormones. Patients complain of tachycardia, weight loss, increased body temperature, tremor, sleep disturbances, and high nervousness. In hypothyroidism, patients have bradycardia, weight gain, decreased body temperature, fatigue, and dry skin. In addition, the hormone calcitonin is produced in the C-cells of the thyroid gland. Calcitonin is mainly involved in calcium balance and lowers short-term calcium in the blood.

Large case-control studies and cohort studies have shown that thyroid disease is a common comorbidity of HS (Table 8) [44,70]. Liakou et al. described that severity of HS was significantly correlated with presence of thyroid disease and active smoking [71]. In a population-based cross-sectional study of 4191 people, the association between HS and thyroid disease has been investigated [72]. There was an increased odds ratio for hypothyroidism (OR 2.91; 95% CI: 2.48–3.40; *p* < 0.001) and hyperthyroidism (OR 2.25; 95% CI: 1.55–3.28; *p* < 0.001) in HS compared to controls. The association between HS and hypothyroidism was also confirmed via multivariate logistic regression analysis. Even after controlling for age, sex, socioeconomic status, and smoking, the association remained significant. The association between HS and hyperthyroidism was not significant in the adjusted model, so HS was only independently significant with hypothyroidism. In contrast to the results of Sherman et al., the study by Miller et al. showed a significant correlation between HS and increased TSH and decreased free T3 [36,72]. Miller et al. retrospectively examined blood of 430 HS patients for thyroid hormones. Clinical hyperthyroidism was also significantly associated after removal of confounding factors (OR of 1.91; 95% CI 1.19–3.07; *p* = 0.02) [36]. HS with vulvar involvement (VHS) is also associated with thyroid disease. López-Llunell et al. studied 25 patients with VHS and found that patients had late onset of disease. The average BMI of VHS was normal (HS with VHS: 23.2 kg/m^2^ vs. HS without: VHS 28.6 kg/m^2^) [73]. A nationwide cohort analysis in Denmark investigated medication of HS patients [74]. No increased hazard ratio for thyroid medication could be found. The authors assume that comorbidities such as thyroid disease occur in severe courses [74]. Gonzoalez-Lopez et al. also reported that thyroid function parameters did not differ between HS patients and controls [75]. Autoimmune antibodies against the thyroid gland also do not differ significantly between HS and non-HS patients [45,75].

### 3.6. Polycystic Ovary Syndrome (PCOS) and HS

Another comorbidity of HS in women is also polycystic ovary syndrome (PCOS). PCOS is an endocrine disorder characterized by hyperandrogenism, cycle disorder, and, less commonly, polycystic ovaries. Many patients demonstrate a phenotype of metabolic syndrome. Pathophysiologically, insulin resistance is present, leading to increased androgen synthesis by IGF-1 in the ovary. Hyperandrogenemia leads to virilization and LH dominance. Patients with PCOS may receive anti-androgenic therapy with metformin or oral contraceptives. The prevalence of PCOS in HS is 9% according to available data. In a meta-analysis with five case-control studies, HS patients were shown to have a 2.64-fold greater risk of PCOS *(*Table 9) [76]. Screening for PCOS can be useful in certain patients.

## 4. Discussion

The role of hormones in HS seems to be complex due to many hormonal feedbacks. Data on sex hormones in HS are controversial, suggesting that androgens are involved in pathogenesis of female HS patients. It is interesting to note that, in female HS patients, genes controlled by androgens are upregulated [13,16]. In men, it seems that androgens play a subordinate role in HS. An investigation of androgens with clinical classification into three subtypes, axillary-mammary, follicular, and gluteal, could be useful for further insights [78]. In Mortimer and coworkers’ study, 88.1% of the patients were of the follicular type and had significantly elevated testosterone [17]. In another study by Harrison et al., no increased testosterone was found, but it is not clear from the data which clinical phenotype was present in these patients [20]. The follicular type of HS shows similarities to acne vulgaris so that increased androgen would be a plausible explanation. To be able to assess this conclusively, clinical phenotyping in further studies is required. It might be useful to investigate whether the follicular type of HS responds better to antiandrogenic therapy than other subtypes. The response to antiandrogenic therapy in women may be related to the segmental AR expression pattern of the skin tunnels [13]. For this reason, it may be useful to investigate the clinical response to antiandrogenic therapy with the AR expression pattern in females. Routine immunohistochemical AR staining could become a tool to extend HS therapy. Antiandrogenic therapy is a good option according to the available data for female patients. The clinical response in HS of spironolactone and finasteride ranged from approximately 40% to 85% [5,25,27,35]. Overall, both drugs are tolerated and few side effects have been reported. Antiandrogenic therapy should not be used in male patients as there is little experience, and it could have more severe side effects. Estrogen does not seem to play a role in pathogenesis of HS [13]. There are hardly any data available on sex hormone progesterone in HS. As some patients demonstrate a link between HS and pregnancy, further investigation of progesterone levels during pregnancy and after pregnancy might be useful. Peripubertal onset of HS, premenstrual deterioration, and postpartum deterioration supported the role of sex hormones in HS [7,11,12]. However, this connection has not yet been proven. A subdivision between hormone-dependent and hormone-dependent HS could be interesting in the future to provide further information, as well as that cross-sex hormone therapy worsens HS, showing that androgens should be considered in HS pathogenesis [18,19].

Insulin resistance is present in between 43.4 and 45.9% of HS patients, which is higher than healthy controls [42,60]. In a cross-sectional and case-control study, IR was significantly increased in HS patients compared to healthy controls (43.4% vs. 16.4%, *p* = 0.001) [60]. Almost half of the patients had insulin resistance according to the data. For this reason, testing for IR in HS patients is useful to detect possible diabetes mellitus at an early stage. This could prevent secondary diseases from diabetes mellitus and metabolic syndrome. Cardiovascular risk factors are significantly more common in HS patients, as also indicated by a significant increase in systemic immune-inflammation-based biomarkers [79,80]. For this reason, typical cardiovascular risk factors should be screened for. The exact mechanism of IR should be investigated in future studies to better understand HS. Antidiabetic therapy with metformin resulted in clinical improvement in 50%–72% of HS patients [64,65,66]. Metformin therapy in HS was well-tolerated. Most common side effects were gastrointestinal symptoms. Metformin causes increased insulin sensitivity and highlights the role of insulin resistance in HS. The results of the double-blind, randomized metformin trial will provide new insights into the hormonal role in HS. It remains to be seen whether HS patients with insulin resistance respond better to metformin therapy. In a case report, a positive effect of liraglutide on HS has been reported. Liraglutide is a GLP-1 analogue and is used in diabetes mellitus. High weight loss and bariatric surgery in HS patients lead to clinical improvement in HS [81]. This supports the fact that impaired glucose tolerance must be considered in HS therapy. Diet also proves beneficial for HS [82]. Ramadan fasting is a well-known diet and provides increased insulin sensitivity [83,84]. A multicenter, observational cross-over pilot study about Ramadan fasting in HS showed significant improvement in IHS4 [82]. Fasting has an impact on adipokine release [85]. The literature clearly indicates that adipokine balance is disturbed in HS. It is shifted towards pro-inflammatory adipokines. The anti-inflammatory adipokine adiponectin is decreased in HS [50,52]. Pro-inflammatory adipokines visfatin, resistin, omentin-1, and RBP4 are elevated in HS. A disbalance in adipokines could lead to impairment in regulation of fat storage glucose tolerance and insulin release [86]. Authors also discuss obesity-induced inflammation in HS, which has an impact on insulin signaling [43].

Thyroid disease and PCOS are also more commonly associated with HS. Both hyperthyroidism and hypothyroidism have been described. The frequent occurrence of thyroid diseases in HS could be related to the smoking behavior of HS patients [36,44,71,72]. Autoimmunity of the thyroid gland is not more common in HS patients [45,70,75]. There is no new research examining the hypothalamic–pituitary axis. However, the current data do not allow any clear conclusions to be drawn about thyroid function in HS. HS with vulva involvement was significantly associated with thyroid disease [73]. The reasons for this association are unknown. PCOS is also common in HS and should be considered if typical symptoms are present. PCOS is characterized by hyperinsulinemia, reduced insulin sensitivity, and hyperandrogenism. Increased insulin causes increased synthesis of androgen in the ovary and adrenal cortex. Other HS patients without PCOS and with insulin resistance could have a similar pathway.

## 5. Conclusions

Overall, there is some evidence that hormones play an important role in HS. However, there is still a lack of randomized controlled trials and experimental studies investigating the influence of hormones in HS. A double-blind randomized metformin trial will provide the first meaningful findings. The influence of insulin resistance on the HS pathomechanisms is currently still underestimated. Future studies should investigate function of adipokines and insulin resistance in HS. A decoupled axis between insulin metabolism, immune system, and hormonal axis could be an important starting point for further HS research.

## Figures and Tables

**Table 1:** Current findings about HS manifestation and exacerbation.

References	Type of Source	Menses	During Pregnancy	Postmenopausal	Conclusion
Vossen et al., 2017 [11]	survey-based cross-sectional study (n = 186; all female)	worsening around menses (43%); no change (53.8%)	worsening (16.7%); no change (53.1%); improvement (30.2%)	no data	There is a significant correlation between perimenstrual worsening and improvement during pregnancy (*p* < 0.01).
Fernandez et al., 2020 [7]	survey in an international HS group (n = 279; all female)	worsening of HS (76.7%); no change (22.2%); improvement (1.1%)	worsening (34.8%); no change (28.7%); improvement (36.6%)	worsening (39.5%); no change (44.2%); improvement (16.6%)	Menstruation causes exacerbation of HS. Pregnancy has mixed effects on HS.
Seivright et al., 2022 [12]	Meta-analyses with 8 studies (n = 672; all female)	no data	worsening (20%); no change (56%); improvement (24%) postpartum deterioration: 60% of patients	no data	During pregnancy, the disease is stable in most cases. After birth, HS worsens in 60% of patients.

**Table 8:** Current findings about thyroid function in HS.

References	Type of Source	Results	Conclusion
Shlyankevich et al., 2014 [44]	retrospective case-control study (n = 1776; 1296 female and 480 male)	− smoking, arthropathies, dyslipidemia, polycystic ovary syndrome, psychiatric disorders, obesity, drug dependence, hypertension, diabetes, thyroid disease, alcohol dependence, and lymphoma were significantly associated with HS (all *p* < 0.01).	HS is associated with thyroid disease.
Gonzoalez-Lopez et al., 2017 [75]	case control study (n = 70; 38 female and 32 male)	− there was no significant difference between thyroid antibodies or thyroid function parameters between HS patients and controls	Autoimmunity of the thyroid gland is not involved in development of HS.
Lee et al., 2018 [45]	case-control study (n = 28,516; 11,036 female and 17,480 male)	− there was no difference in the prevalence of Hashimoto’s thyroiditis or Grave’s disease in HS (*p* = 0,881 und *p* = 0.250)	Autoimmunity of the thyroid gland is not involved in development of HS.
Miller et al., 2018 [36]	retrospective comparative cross-sectional study (n = 430; 292 female and 138 male)	− an age- and sex-adjusted analysis showed a significantly lower value (*p* < 0.001) for TSH and a significantly higher value (*p* < 0.0001) of free T3. − there was also a significant association between clinical hyperthyroidism and HS with an OR of 1.91 (95% CI 1.19–3.07; *p* = 0.02; adjusted for the factors BMI, smoking, and oral contraception)	HS is associated with hyperthyroidism.
Kimball et al., 2018 [70]	retrospective matched cohort design (n = 5357; 3873 female and 1484 male)	− prevalence of thyroid disease is increased (HS-mild vs. no HS: 12.0 vs. 8.6%; HS-severe vs. no HS: 12.0 vs. 7.8%) − no significant association between Hashimoto’s thyroiditis and HS	HS is associated with thyroid disease.Autoimmunity of the thyroid gland is not involved in development of HS.
Sherman et al., 2021 [72]	cross-sectional large-scale population-based study (n = 4191; 2590 female and 1601 male)	− Odds ratio for hypothyroidism was 2.91 (95% CI 2.48–3.40; *p* < 0.001) and for hyperthyroidism 2.25 (95% CI 1.55–3.28; *p* < 0.001) − association between HS and hyperthyroidism was not significant in the adjusted model.	HS is independently associated with hypothyroidism.
Liakou et al., 2021 [71]	prospective cross-sectional single-center study (n = 290; 248 female and 242 male)	− In a logistic regression model, active smoking and thyroid disease were associated with disease severity Hurley (OR 1.38; 95% CI 1.11–1.65 and OR 1.30; 95% CI 1.09–1.76) and according to the IHS4 score system (OR 1.23; 95% CI 1.09–1.64 and OR 1.42; 95% CI 1.19–1.66)	Thyroid disorders and active smoking are significantly associated with a higher HS stage.
López-Llunell et al., 2021 [73]	clinical cross-sectional study (n = 25; all female)	− a significant positive association was found between HS with vulva involvement (VHS) and fistulas (*p* < 0.001), acne vulgaris (*p* = 0.021), and thyroid disease (*p* = 0.006) − there was a negative association between VHS and axillary lesions (*p* = 0.001) − VHS patients had a later mean age of onset and a lower body mass index (BMI) (*p* = 0.035; *p* = 0.048) than those without vulvar involvement.	VHS is significantly associated with later onset, lower BMI, and thyroid disease.
Andersen et al., 2021 [74]	prospective survival analysis on a nationwide cohort of blood donors (n = 40; 23 female and 17 male)	− hazard ratio in HS has been studied based on prescribing medicines − no higher HR of antidiabetic or thyroid drugs could be detected (*p* = 0.084; *p* = 0.35) − most of the patients included had a mild course of the disease	Comorbidities (e.g., diabetes, thyroid disease) may occur first in severe disease or later in the course of the disease.

**Table 9:** Current findings on PCOS and HS.

References	Type of Source	Results	Conclusion
Garg et al., 2018 [77]	case-control study (n = 2090; all female)	− prevalence of PCOS in HS was higher than in patients without HS (9.0% vs. 2.9%; *p* < 0.0001). − probability of patients with HS having PCOS was 2.14-fold higher (95% CI 2.04–2.24) than in patients without HS	HS is associated with PCOS.
Phan et al., 2020 [76]	Meta-analyses with 5 case-control studies (n = 20,532; all female)	− probability of patients with HS having PCOS was 2.64-fold higher (95% CI 1.69–4.11; *p* < 0.00001) than in patients without HS	There is a link between HS and PCOS. HS patients with evidence of hyperandrogenism may benefit from screening for PCOS and from antiandrogen therapy.

## Data Availability

Not applicable.
